# Remote electroacupuncture conditioning protects the heart via fine-tuning the dual role of the SDF-1α/CXCR4 axis in myocardial ischemia–reperfusion injury

**DOI:** 10.1186/s13020-026-01470-5

**Published:** 2026-08-18

**Authors:** Senlei Xu, Jianfeng Lian, Shiyu Chen, Yan Zuo, Hui Xiong, Ying Lin, Yihuang Gu, Hongru Zhang

**Affiliations:** 1 School of Acupuncture and Tuina, School of Regimen and Rehabilitation, Nanjing University of Chinese Medicine, Nanjing, China; 2 School of Integrated Traditional Chinese and Western Medicine, Fujian University of Traditional Chinese Medicine, Fuzhou, China; 3 Jiangsu Province Engineering Research Center of Traditional Chinese Medicine Health Preservation, Nanjing University of Chinese Medicine, Nanjing, China; 4 Department of Acupuncture, Haian Hospital of Chinese Medicine Affiliated to Nanjing University of Chinese Medicine, Nantong, China; 5 Key Laboratory of Acupuncture and Medicine Research of Ministry of Education, Nanjing University of Chinese Medicine, Nanjing, China; 6 School of Integrative Medicine, Nanjing University of Chinese Medicine, Nanjing, China; 7 School of Traditional Chinese Medicine, Nanjing, China; 8 Dan’an College, Nanjing University of Chinese Medicine, Nanjing, China

**Keywords:** Cardioprotection, MIRI, Electroacupuncture, SDF-1α/CXCR4 pathway, Neutrophil

## Abstract

**Background:**

Remote electroacupuncture (EA) conditioning on forearm protects against myocardial ischemia–reperfusion injury (MIRI). However, the cardioprotective mechanisms of RIC have not been fully elucidated. This study aimed to investigate whether EA could balance the pro-survival and pro-inflammatory responses in MIRI rats via SDF-1α/CXCR4 axis.

**Methods:**

SD rats were subjected to MIRI and underwent EA treatment before reperfusion therapy. Infarct size, cardiac function, and neutrophil infiltration were evaluated after 24-h of reperfusion. The mRNA and protein levels of SDF-1α in the myocardial border and infarct zones, were measured by RT-qPCR and Western blotting to reveal its spatial distribution. We examined serum SDF-1α levels and its myocardial scavenger receptor CXCR7 expression to elucidate the mechanism underlying its spatial distribution. Then, CXCR4/ERK pathway was evaluated at 15 min and 24 h after EA to examine the time-dependent dual effects of SDF-1α on CXCR4.

**Results:**

Our results showed that EA attenuated infarct size and mortality, improved cardiac function after 24 h of MIRI. We identify that SDF-1α is a key humoral factor mediating EA-induced cardioprotection. In ischemic myocardium, the infarct zone exhibited a higher SDF-1α concentration than the border zone following EA treatment, a difference potentially linked to the elevated expression of CXCR7 in the border zone. In the early phase of reperfusion (15 min), our study showed that EA promoted the expression of the CXCR4 receptor, which in turn triggered ERK activation. This rapid ERK activation via SDF-1α/CXCR4 promoted cardiomyocyte survival and attenuated reperfusion-induced apoptosis. While at 24 h post-reperfusion, saturating signals of SDF-1α by EA downregulated CXCR4 expression, along with phosphorylation of its downstream effectors ERK and NF-κB p65. This coordinated downregulation facilitates inflammatory resolution by promoting neutrophil apoptosis.

**Conclusions:**

Cardioprotection by EA can be mediated by humoral factors SDF-1α that accumulate in infarct myocardium. SDF-1α triggers pro-survival CXCR4/ERK signaling at 15 min of reperfusion, whereas it promotes neutrophil apoptosis via CXCR4 degradation to facilitate inflammatory resolution by 24 h, thereby fine-tuning the dual role of the SDF-1α/CXCR4 axis in MIRI.

## Introduction

Acute myocardial infarction (AMI) is a leading cause of death worldwide and the most common etiology of chronic heart failure [1]. Timely reperfusion remains the gold standard of care in AMI patients but paradoxically results in additional myocardial damage, a phenomenon termed MIRI [2]. For approximately three decades, it has been known that remote ischemic conditioning (RIC), induced non-invasively by brief cycles of limb ischemia and reperfusion, confers protection against reperfusion injury [3]. The original method of inducing RIC manually with pneumatic limb cuffs posed challenges for long-term compliance. Subsequent research revealed that the benefits could be replicated via non-ischemic stimuli, with remote EA conditioning emerging as a promising green therapy that provides a facile, non-invasive alternative for achieving remote conditioning, as evidenced by its ability to ameliorate myocardial injury and enhance cardiac function [4,5].

Remote conditioning involves the release of a humoral factor(probably a peptide > 3.5 kDa and < 15 kDa), which is carried by the blood to the remote target heart against MIRI [6]. Stromal cell derived factor-1α (SDF-1α), an 8 kDa CXC chemokine, was found to be released into the plasma of rats following RIC on a single limb [7]. Importantly, EA at the PC6 acupoint significantly elevates SDF-1α levels in the ischemic myocardium of AMI rats, thereby producing a cardioprotective effect [8]. SDF-1α is able to directly protect the isolated heart from acute injury, by binding to its receptor CXCR4 [9]. In addition to the acute effect, many studies have investigated its potential to recruit inflammatory and stem cells to the ischemic heart in order to aid tissue clearance and healing [10].

However, investigations into the therapeutic targeting of the SDF-1α/CXCR4 axis against MIRI have produced conflicting evidence. Direct intracardiac injection of SDF-1α protein in mice reduced infarct size and improved cardiac function at 4 weeks post-MI [11–13]. In contrast, injecting SDF-1α into the border zone at 2 weeks post-MI impaired cardiac function [14]. Similarly, continuous inhibition of CXCR4 with AMD3100 after MI worsened cardiac outcomes, whereas a single dose preserved cardiac function [15,16]. In fact, SDF-1α can tilt the balance of CXCR4 signaling toward survival or inflammation in a cell-type-specific manner; consequently, the net outcome of modulating this axis is spatiotemporally determined by the intervention context within the healing infarct [17].

In our study, we identified that SDF-1α acts as a key humoral signal transmitted to the heart in response to remote EA conditioning applied to the forearm following 24 h of MIRI. However, the cardioprotective mechanisms of EA via the SDF-1α/CXCR4 axis have not been fully elucidated. This study aimed to investigate whether EA can modulate the CXCR4 signaling to balance the pro-survival and pro-inflammatory responses against reperfusion injury.

## Materials and methods

### Experimental animals

Healthy specific pathogen-free (SPF) male Sprague–Dawley (SD) rats, 8 weeks old, weighing 260 ± 20 g were supplied by Vital River Laboratory Animal Technology Co., Ltd. (License No.: SCX (Zhe) 2019-0001). The animals were housed under standard conditions with a 12-h light/dark cycle, a temperature of 25 ± 2 °C, and a relative humidity of 50 ± 5%, and were provided with a standard diet. All experimental procedures were approved by the Animal Ethics Committee of Nanjing University of Chinese Medicine (Ethical Approval No.202005A030), Clinical trial number: not applicable, and all experimental procedures conformed to the Guide for the Care and Use of Laboratory Animals by the National Institutes of Health.

### MIRI model

Following the induction of anesthesia with 5% isoflurane/O₂ (0.4 L/min), rats showing a loss of righting reflex were positioned supine on a thermostatic plate (37 ± 3 °C), intubated, and ventilated. Anesthesia was maintained with 2% isoflurane (0.4 L/min O₂) at 60–70 breaths/min. After surgical site preparation, a longitudinal thoracotomy exposed the heart for LAD ligation (using 6–0 silk suture) between the pulmonary artery and left atrial appendage to induce ischemia.

Successful MIRI modeling was confirmed by ischemic signs (ventricular wall hypokinesis, pallor/cyanosis, ST elevation) and subsequent reperfusion-induced reddening of the affected area.

### Experimental design

**Fig. 1:**
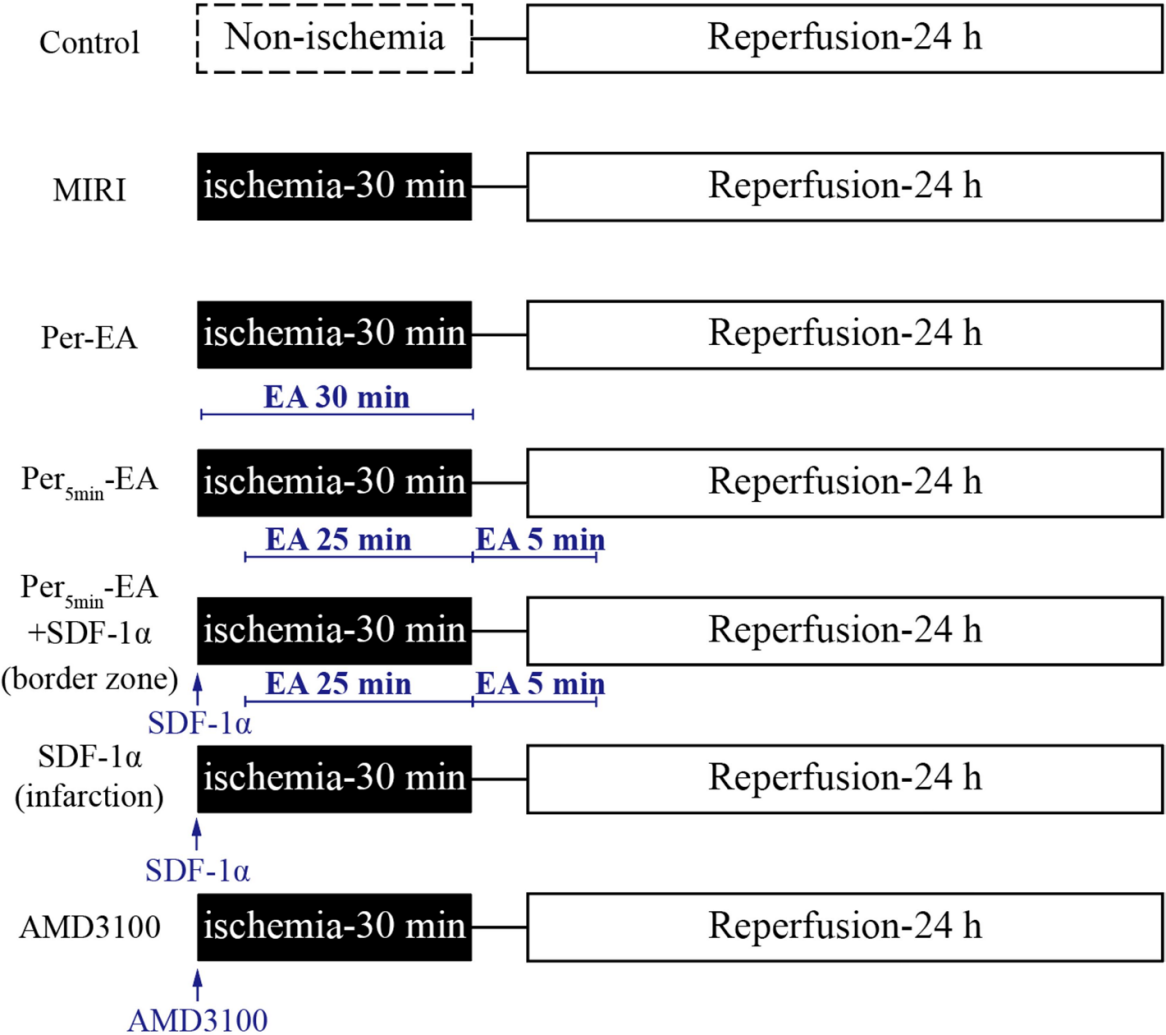
A schematic illustration of the experimental design. The overall animal study design used to test the cardioprotection of remote electroacupuncture conditioning in a rat model of MIRI

The experimental design is illustrated in Fig. 1. After 3 days of adaptive feeding, the rats were randomly assigned using a random number table into 4 groups in the first phase: the Control group (Control), MIRI model group (MIRI), the Per-conditioning electroacupuncture group (Per-EA) group and the Per-conditioning electroacupuncture after 5 min of ischemia (Per_5min_-EA) group, the last of which was added based on the research findings.

In the second phase, rats were randomly assigned using a random number table into 3 groups: Per_5min_-EA + SDF-1α (border zone) group: Underwent thoracotomy, followed by three injections of SDF-1α (1 μg/rat) into the border zone of the LAD perfusion area below the left atrial appendage. All other procedures were consistent with those in the Per_5min_-EA group. The SDF-1α (infarction) group: Underwent thoracotomy, followed by an injection of SDF-1α (1 μg/rat) into the center of the LAD perfusion area. Five minutes later, all other procedures were consistent with MIRI group. AMD3100 group: Received an intraperitoneal injection of AMD3100 (5 mg/kg), and all other procedures were consistent with MIRI group.

### Electroacupuncture treatment

PC6 (Neiguan) was located at medial radioulnar interspace at the distal 1/6 forearm point). Bipolar electroacupuncture was administered via perpendicular insertion (0.5–0.8 cm depth) of disposable needles (0.2 mm × 25 mm) connected to a HANS device (HANS, China). Stimulation used our laboratory's optimized parameters: 2 mA intensity, 2/100 Hz alternating frequency, for 30 min.

### Reagent injection

According to the manufacturer’s instructions, 10 mg of AMD3100 powder was dissolved in 1 mL of ethanol and sonicated to mix thoroughly. This solution was then added to 19 mL of physiological saline, followed by sonication to obtain a homogeneous solution at a concentration of 0.5 mg/mL. The drug was administered via intraperitoneal injection at a dosage of 5 mg/kg.

Recombinant rat SDF-1α (100 μg) was reconstituted in 1 mL of ultrapure water, vortexed thoroughly, and prepared as a 0.1 mg/mL solution. A dose of 1 μg per rat was administered via intracardiac injection.

### Cardiac ultrasound

After 24 h of reperfusion, transthoracic echocardiography was performed on rats from each group using an ESAOTE animal Doppler ultrasound imaging system. Anesthesia was induced with 5% isoflurane in pure oxygen and maintained with 2% isoflurane in pure oxygen. Chest hair was removed with an electric clipper. Using the long-axis view of the sternum, left ventricular wall motion was recorded via M-mode tracing. Left ventricular end-systolic diameter (LVESD) and left ventricular end-diastolic diameter (LVEDD) were measured. Left ventricular ejection fraction (LVEF) and fractional shortening (LVFS) were calculated using the following formulas:$LVEF \, = \, \left[ {\left( {LVEDV \, - \, LVESV} \right) \, / \, LVEDV} \right] \, \times \, 100\% ; \, LVFS \, = \, \left[ {\left( {LVEDD \, - \, LVESD} \right) \, / \, LVEDD} \right] \, \times \, 100\% .$

### Evans blue-TTC dual staining and infarct size measurement

After 24 h of reperfusion, the LAD was re-ligated via an abdominal thoracotomy. Ice-cold Evans blue dye (2–3 mL) was injected retrograde from the root of the exposed inferior vena cava. Following a 3-min staining period, the heart was excised, frozen at –20 °C for 30 min, and sectioned transversely into five 2 mm slices from apex to base. Sections were incubated in 1% TTC at room temperature for 2–3 min, then transferred to a 37 °C oven for 15 min. After fixation with 4% paraformaldehyde, slices were imaged. Myocardial tissue exhibited three distinct regions: blue (normal viable myocardium), non-blue area at risk (AAR), and within the AAR, brick-red (viable) and pale white (necrotic, AON) areas. AAR% (AAR/total area × 100%) and AON% (AON/AAR × 100%) were quantified using ImageJ 2.1.

### H&E staining and immunofluorescence

After 24 h of reperfusion, cardiac tissue distal to the ligation site was collected, fixed in 4% paraformaldehyde, embedded in paraffin, and sectioned at a thickness of approximately 5 μm. The sections were stained with hematoxylin and eosin (H&E), dehydrated, mounted, and examined under an optical microscope (Olympus, Japan) to assess inflammatory cell infiltration.

For immunofluorescence, paraffin sections were deparaffinized, rehydrated, and subjected to antigen retrieval in EDTA buffer using microwave heating. After washing with PBS, sections were blocked with 5% BSA for 20 min. The sections were then incubated with two primary antibodies overnight at 4 °C. Following three PBS washes, they were incubated with two corresponding secondary antibodies for 1 h at room temperature, protected from light. After additional PBS washes, nuclei were counterstained with DAPI for 10 min in the dark. Following a final wash, sections were mounted and observed under a laser scanning confocal microscope.

### Enzyme-linked immunosorbent assay (ELISA)

Following 24 h of reperfusion, blood samples were collected from the abdominal aorta. After high-speed centrifugation under low-temperature conditions (4 °C, 3600 rpm, 15 min), the supernatant serum was collected. Serum concentrations of cTnI and CK-MB were measured according to the instructions provided with the respective commercial ELISA kits.

### Western blot

Total protein was extracted from myocardial tissue using RIPA lysis buffer. Protein concentration was determined with a BCA protein assay kit. Protein samples were separated by SDS-PAGE and transferred to PVDF membranes. After blocking with 5% BSA for 1 h, the membranes were incubated with primary antibodies at 4 °C overnight. The following day, membranes were washed with TBST and incubated with secondary antibodies at room temperature for 2 h. Protein bands were visualized using a chemiluminescence reagent and imaged with a gel documentation system for analysis.

### Quantitative real-time PCR (qRT-PCR)

Total RNA was extracted from myocardial tissue with Trizol reagent and reverse-transcribed into cDNA. qRT-PCR was performed to amplify the target genes under the following conditions: pre-denaturation at 95 °C for 10 min (1 cycle); followed by 40 cycles of denaturation at 95 °C for 15 s and annealing/extension at 60 °C for 20 s. The primer sequences used were as follows:

**SDF-1α:**

Forward: 5′-ACAAAGCCTTAAACAAGAGGCT-3′

Reverse: 5′-GGAGCAGAGGAAGTGGCTAT-3′

**iNOS:**

Forward: 5′-AAGAGGAACAACTACTGCTG-3′

Reverse: 5′-ATTTCTTCAGAGTCTGCCC-3′

**CD206:**

Forward: 5′-TACACAAATTCAGGGTTCTGG-3′

Reverse: 5′-GATGCTGCTATTATGTCGCT-3′

**Arg1:**

Forward: 5′-ATGTGCCCTCTGTCTTTTAGG-3′

Reverse: 5′-GTCCTGAAAGTAGCCCTGTCT-3′

**MPO:**

Forward: 5′-TGAATCCTCGCTGGAATGG-3′

Reverse: 5′-GGTATGTGATGATCTGGACCA-3′

**IL-1β:**

Forward: 5′-TTCATCTTTGAAGAAGAGCCC-3′

Reverse: 5′-CACTTGAACTGGGTACTGG-3′

**TNF-α:**

Forward: 5′-CTCTAATCAGCCCTCTGGC-3′

Reverse: 5′-GAGGGTTTGCTACAACATGG-3′

**GAPDH:**

Forward: 5′-TTGTGCAGTGCCAGCCTC-3′

Reverse: 5′-ACCAGCTTCCCATTCTCAGC-3′

GAPDH was used as the internal control, and relative gene expression was calculated using the 2^^(−ΔΔCT)^ method.

### Statistical analysis

Statistical analysis was performed using SPSS software (version 23.0; IBM, USA). Measurement data are presented as mean ± standard deviation (X̄ ± SD). For comparisons among groups, one-way analysis of variance (ANOVA) was applied when data followed a normal distribution and homogeneity of variance assumptions were met, with post-hoc pairwise comparisons conducted using Bonferroni's test. If the assumptions of normality or homogeneity of variance were violated, the Kruskal–Wallis H test was used. A P-value < 0.05 was considered statistically significant.

## Results

### Remote EA perconditioning decreases infract size and improves cardiac function in MIRI rats

**Fig. 2:**
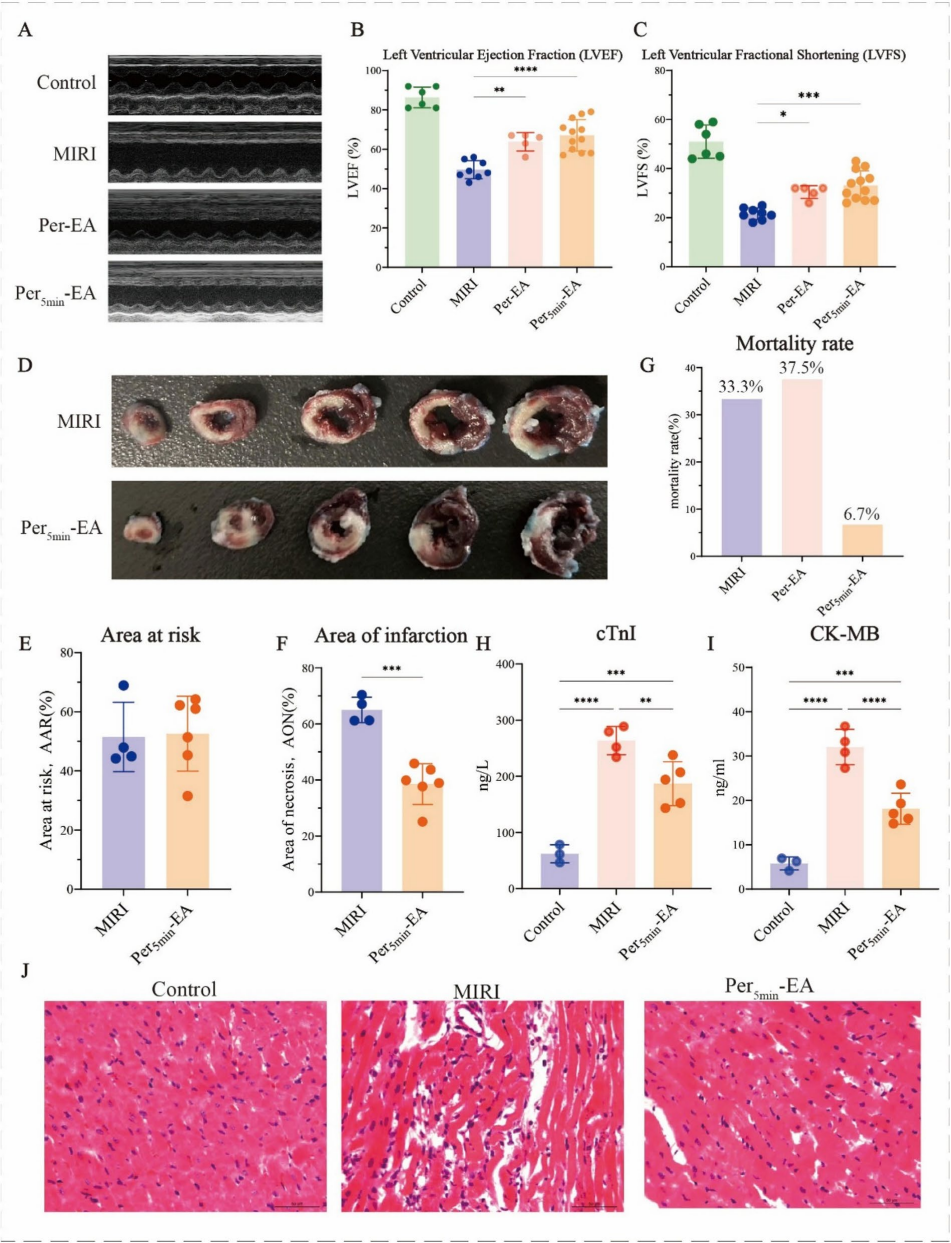
Remote EA Perconditioning Decreases Infract Size and Improves Cardiac Function in MIRI Rats. **A** Representative M-mode echocardiograms showing left ventricular wall motion. **B**, **C** Measurement of LVEF and LVFS 24 h after MIRI, n = 5–12. **D** Representative Evans blue–TTC dual-stained sections of rat myocardium. Blue areas indicate non-ischemic tissue; non-blue areas represent the area at risk (AAR), which is further divided into white ischemic infarct tissue and red viable myocardium. **E**, **F** Comparison of AAR and infarct size among groups 24 h after MIRI, n = 4–7. **G** Mortality rate 24 h after MIRI. **H**, **I** Serum cTnI and CK-MB levels measured by ELISA after 24 h of MIRI, n = 3–5. **J** H&E staining of myocardial tissues 24 h after MIRI (× 400). All data are presented as mean ± SD. *p < 0.05, **p < 0.01, ***p < 0.001, ****p < 0.0001

To investigate whether EA reduces infarct size and improves cardiac function, we administered remote electroacupuncture conditioning to rats after ischemia but prior to reperfusion. At 24 h after MIRI, the MIRI model group (MIRI) exhibited LVEF and LVFS values of 49.63 ± 4.60% and 21.63 ± 2.39%, respectively. Both the Per-EA and Per_5min_-EA groups showed significant improvements in cardiac function, with LVEF values of 63.8 ± 4.66% and 61.92 ± 7.33%, and LVFS values of 30.40 ± 2.61% and 29.42 ± 4.91%, respectively (Fig. 2A-C). The difference between the two treatment groups was not statistically significant. Additionally, initial mortality rates were 33.3% (MIRI group) and 37.5% (Per-EA group), with most Per-EA deaths coinciding with EA application in the first 5 min of ischemia. Therefore, a modified Per_5min_-EA group received EA starting 5 min post-ischemia, which achieved a much lower mortality rate of 6.7% (Fig. 2G). These results indicate Per_5min_-EA effectively alleviated cardiac dysfunction and reduced mortality rate following MIRI.

We next evaluated the acute cardioprotective effect of EA against MIRI by Evans blue–TTC dual staining. As shown in Fig. 2D, E, no significant difference was observed in the area at risk (AAR, non-blue area) among the groups, indicating consistent and stable ischemic insult. Further quantification of infarct size (Fig. 2D, F) revealed that the Per_5min_-EA group exhibited a significant reduction in infarct area compared with the MIRI group. Serum levels of the key myocardial injury markers CK-MB and cTnI, which were significantly elevated 24 h post-MIRI, showed a significant reduction in the Per_5min_-EA group compared to the MIRI group ​(Fig. 2H, I). H&E staining of cardiac tissue revealed distinct morphological changes across groups after 24 h of reperfusion ​(Fig. 2J). The MIRI group exhibited​ disrupted tissue architecture, including broken or dissolved fibers, enlarged intermuscular spaces, inflammatory cell infiltration, and vacuolar degeneration in the infarcted area. Notably, the Per_5min_-EA group showed​ marked improvement in myocardial fiber organization and reduced inflammatory infiltration. These results strongly indicated that remote electroacupuncture conditioning attenuates the deterioration of left ventricular function in MIRI.

### Per_5min_-EA attenuates inflammatory response in infarcted myocardium after 24 h of MIRI by promoting neutrophil apoptosis

**Fig. 3:**
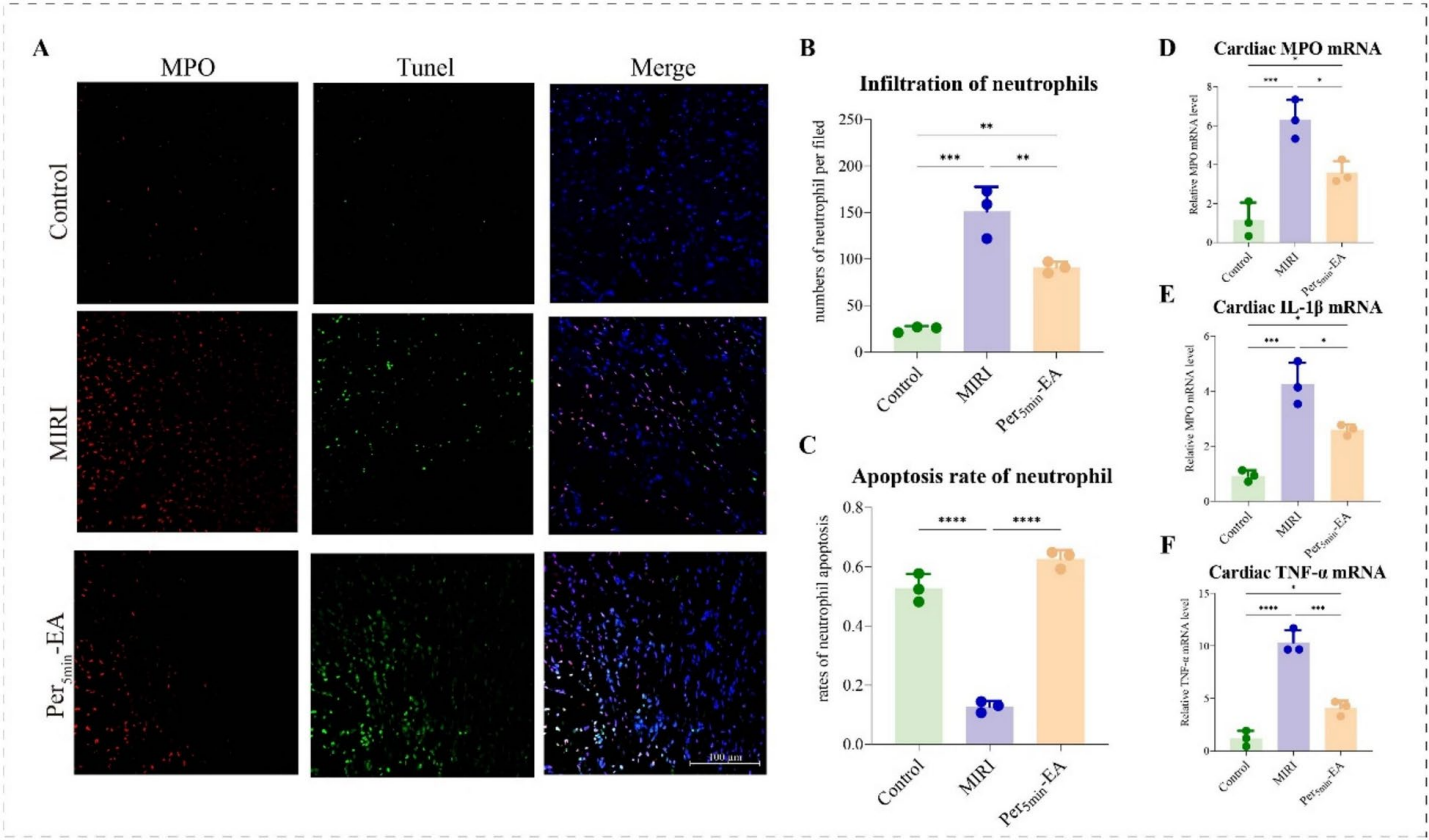
Per_5min_-EA alleviates the inflammatory response in the infarcted myocardium. **A** MPO immunofluorescence staining showing neutrophil infiltration in the infarcted myocardium; MPO and TUNEL co-staining identifying apoptotic neutrophils. Red: MPO; green: TUNEL; blue: DAPI. Magnification: × 40. Scale bar = 100 μm. **B** Number of MPO⁺ cells, representing neutrophil accumulation, n = 3. **C** Proportion of MPO⁺ TUNEL⁺ cells, indicating apoptotic neutrophils. Apoptosis rate = (number of MPO⁺TUNEL⁺ cells)/(number of MPO⁺ cells), n = 3. **D**–**F** Gene expression levels of MPO, IL-1β, and TNF-α in the infarcted myocardium after 24 h of MIRI,n = 3, All data are presented as mean ± SD. *p < 0.05, **p < 0.01, ***p < 0.001, **** p < 0.0001

Neutrophil infiltration is a hallmark of inflammatory injury following MIRI and exerts detrimental effects on the heart; conversely, depletion of neutrophils has been shown to reduce infarct size and attenuate myocardial injury [18,19]. Therefore, we assessed neutrophil infiltration at after 24 h of MIRI by immunofluorescence. The results showed a significantly higher number of MPO⁺ signals in the MIRI group compared to the Control group, indicating substantial neutrophil accumulation in the infarcted myocardium after MIRI. In contrast, Per_5min_-EA treatment markedly reduced local neutrophil infiltration (Fig. 3A, B).

Furthermore, we performed MPO and TUNEL co-staining to distinguish apoptotic neutrophils. We found that the Per_5min_-EA group exhibited a significantly higher rate of neutrophil apoptosis compared to the MIRI group (Fig. 3C). Apoptotic neutrophils can initiate inflammation resolution and suppress further neutrophil recruitment, thereby mitigating myocardial injury [20,21]; delayed neutrophil apoptosis, on the other hand, has been associated with worsened clinical outcomes in heart failure patients [22]. In addition, gene expression levels of the pro-inflammatory cytokines MPO, IL-1β, and TNF-α were elevated in the infarcted myocardium after 24 h of MIRI, but were significantly suppressed by Per_5min_-EA treatment (Fig. 3D-F). Together, these results demonstrate that Per_5min_-EA alleviates the inflammatory response in the infarcted myocardium after 24 h of MIRI by promoting neutrophil apoptosis.

### SDF-1α upregulation in the infarcted myocardium is associated with the cardioprotective effect of Per_5min_-EA

**Fig. 4:**
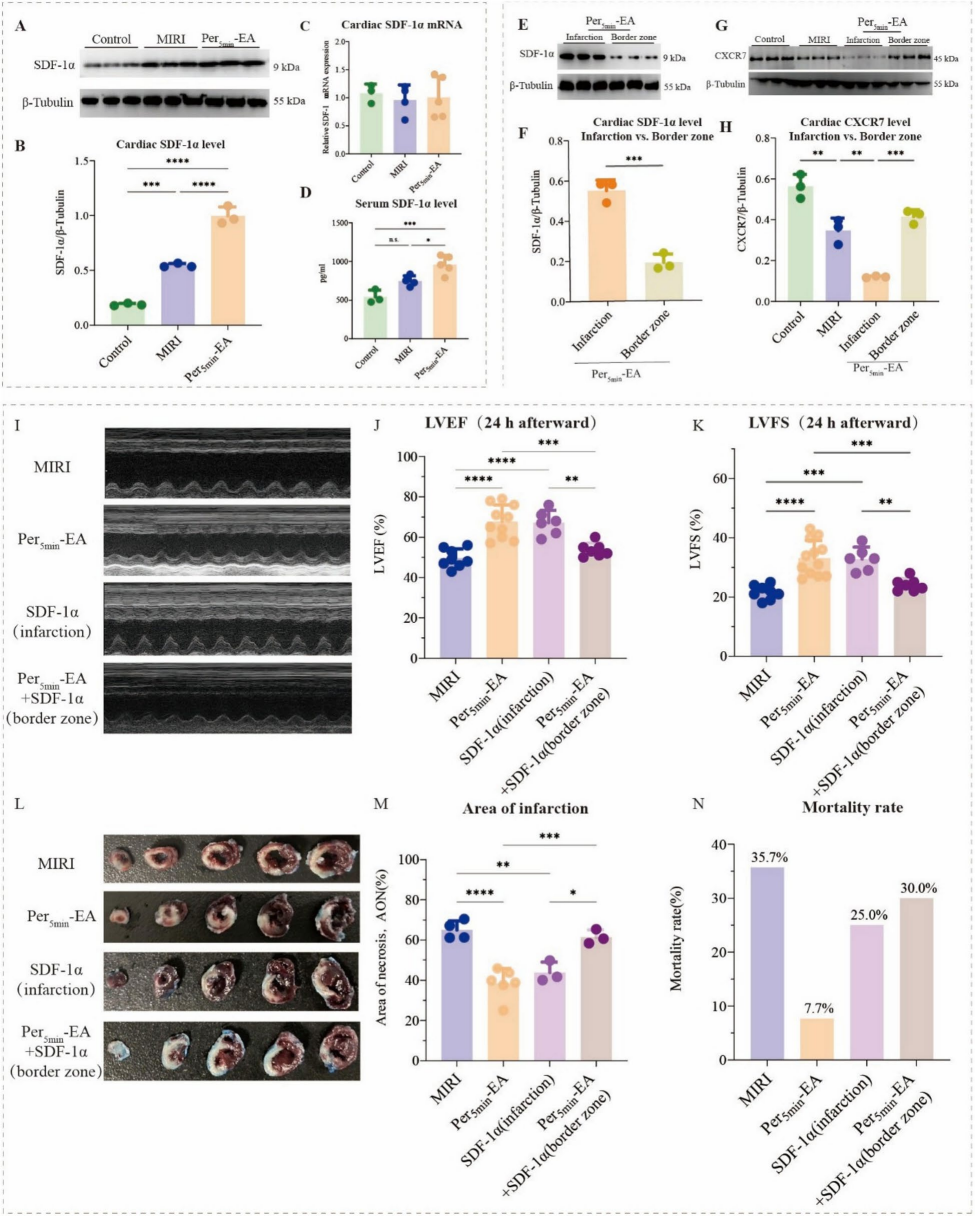
Per_5min_-EA promotes the expression and localized distribution of SDF-1α in the infarcted myocardium to provide cardioprotection. **A** SDF-1α protein expression in the infarcted myocardium was detected by Western blot after 24 h of MIRI. **B** The quantitative analysis of SDF-1α expression normalized to β-Tubulin, n = 3. **C** SDF-1α mRNA expression in the infarcted myocardium after 24 h of MIRI, measured by RT-qPCR, n = 3–5. **D** Serum SDF-1α levels 24 h after MIRI, determined by ELISA, n = 3–5. **E**, **F** Protein levels of SDF-1α in the infarct zone versus the border zone 24 h after MIRI, assessed by Western blot, n = 3. **G**, **H** Protein levels of CXCR7 in the infarct zone versus the border zone 24 h after I/R, assessed by Western blot, n = 3. **I** Representative M-mode echocardiograms showing left ventricular wall motion 24 h after MIRI. **J**, **K** LVEF and LVFS measurements from echocardiographic analysis, n = 5–10. **L** Representative TTC-Evans blue-stained cross-sections of the left ventricle 24 h after MIRI. Blue: non-ischemic tissue; non-blue: area at risk (AAR); white: necrotic area (AON); red: ischemic but viable myocardium. **M** Infarct size expressed as a percentage of the AAR (AON/AAR × 100%), n = 3–6. **N** Mortality rates 24 h after MIRI. All data are presented as mean ± SD. *p < 0.05, **p < 0.01, ***p < 0.001, **** p < 0.0001

Previous studies have shown that SDF-1α promotes neutrophil recruitment while suppressing their infiltration after ischemic injury, thereby reducing myocardial damage and infarct size [13,23]. Therefore, we measured SDF-1α expression in the infarcted myocardium using Western blotting. The results showed that SDF-1α expression was increased in the MIRI group after 24 h of MIRI compared with the Control group. Furthermore, Per_5min_-EA treatment further upregulated SDF-1α expression, which was significantly higher than that in the MIRI group (Fig. 4A, B), suggesting that SDF-1α may be associated with the cardioprotective effect of Per_5min_-EA.

### Per_5min_-EA promotes localized SDF-1α accumulation via dual regulation of extracardiac recruitment and intracardiac degradation

To determine whether the increased myocardial SDF-1α was transcriptionally regulated, we measured SDF-1α mRNA levels using qRT-PCR. No significant differences in SDF-1α gene expression were observed among the groups (Fig. 4C), suggesting that SDF-1α was not synthesized locally in the myocardium. We then quantified serum SDF-1α levels by ELISA. The results showed elevated SDF-1α in the MIRI group, which was further increased by Per_5min_-EA treatment (Fig. 4D).

Previous studies have reported that cerebral ischemia induces a focused distribution of SDF-1α centered on the infarct zone [24,25]. This pattern is attributed to decreased expression of CXCR7–a scavenger receptor that internalizes and degrades SDF-1α–in the infarcted brain region, thereby maintaining high SDF-1α levels in the core infarct while reducing its concentration in the border zone, ultimately establishing an SDF-1α concentration gradient [24]. To investigate whether a similar gradient exists in the infarcted myocardium, we compared SDF-1α expression between the infarct zone and the border zone within heart. SDF-1α levels were significantly higher in the infarct zone than in the border zone (Fig. 4E, F).

To further examine whether the SDF-1α gradient in myocardial infarction was associated with CXCR7, we compared CXCR7 expression between the two regions. CXCR7 expression in the infarct zone was significantly lower in the MIRI group than in the Control group, and Per_5min_-EA further reduced infarct-zone CXCR7 levels. Moreover, in the Per_5min_-EA group, CXCR7 expression in the infarct zone was significantly lower than that in the border zone (Fig. 4G, H).

These findings indicate that Per_5min_-EA enhances serum SDF-1α levels and promotes localized accumulation of SDF-1α in the infarcted myocardium by suppressing CXCR7-mediated internalization and degradation, thereby regulating SDF-1α distribution through both extracardiac recruitment and intracardiac stabilization.

### Focal SDF-1α distribution in the infarct zone underlies the cardioprotection of Per_5min_-EA

To further confirm that the focal distribution of SDF-1α in the infarct zone is a key factor in the cardioprotective effect of Per_5min_-EA, we directly injected SDF-1α into the infarct zone and evaluated its functional impact. The results showed that, compared with the MIRI group, SDF-1α (infarction) group increased LVEF and LVFS at 24 h after MIRI to 67.2 ± 6.83% and 32.8 ± 4.49%, respectively–a level of protection that was not statistically different from that observed in the Per_5min_-EA group (Fig. 4I–K). We next investigated whether disrupting the focal distribution of SDF-1α could eliminate the protective effect of Per_5min_-EA. By injecting SDF-1α into the border zone to disrupt its gradient, while maintaining the same EA protocol, we found that cardiac function of Per_5min_-EA + SDF-1α (border zone) group was significantly lower than in the Per_5min_-EA group, with LVEF and LVFS values of 53.7 ± 3.35% and 24.1 ± 2.12%, respectively (Fig. 4I–K).

Infarct size was also compared among groups 24 h after MIRI. No significant differences were observed in the area at risk (AAR), indicating consistent ischemic injury and reproducible MIRI model establishment across groups. Further analysis of the area of necrosis (AON) revealed that SDF-1α (infarction) group reduced AON to 40.7 ± 8.83%, an effect comparable to that of Per_5min_-EA. In contrast, the Per_5min_-EA + SDF-1α (border zone) group exhibited a significantly larger AON (61.5 ± 3.57%) than the Per_5min_-EA group (Fig. 4L, M). In addition, both Per_5min_-EA and SDF-1α (infarction) treatment reduced mortality compared with the MIRI group (Fig. 4N). These results further confirmed that the focal distribution of SDF-1α in the infarct zone is a critical mechanism underlying the cardioprotective effect of Per_5min_-EA.

### CXCR4, the receptor for SDF-1α, is downregulated by Per_5min_-EA in the infarcted myocardium

**Fig. 5:**
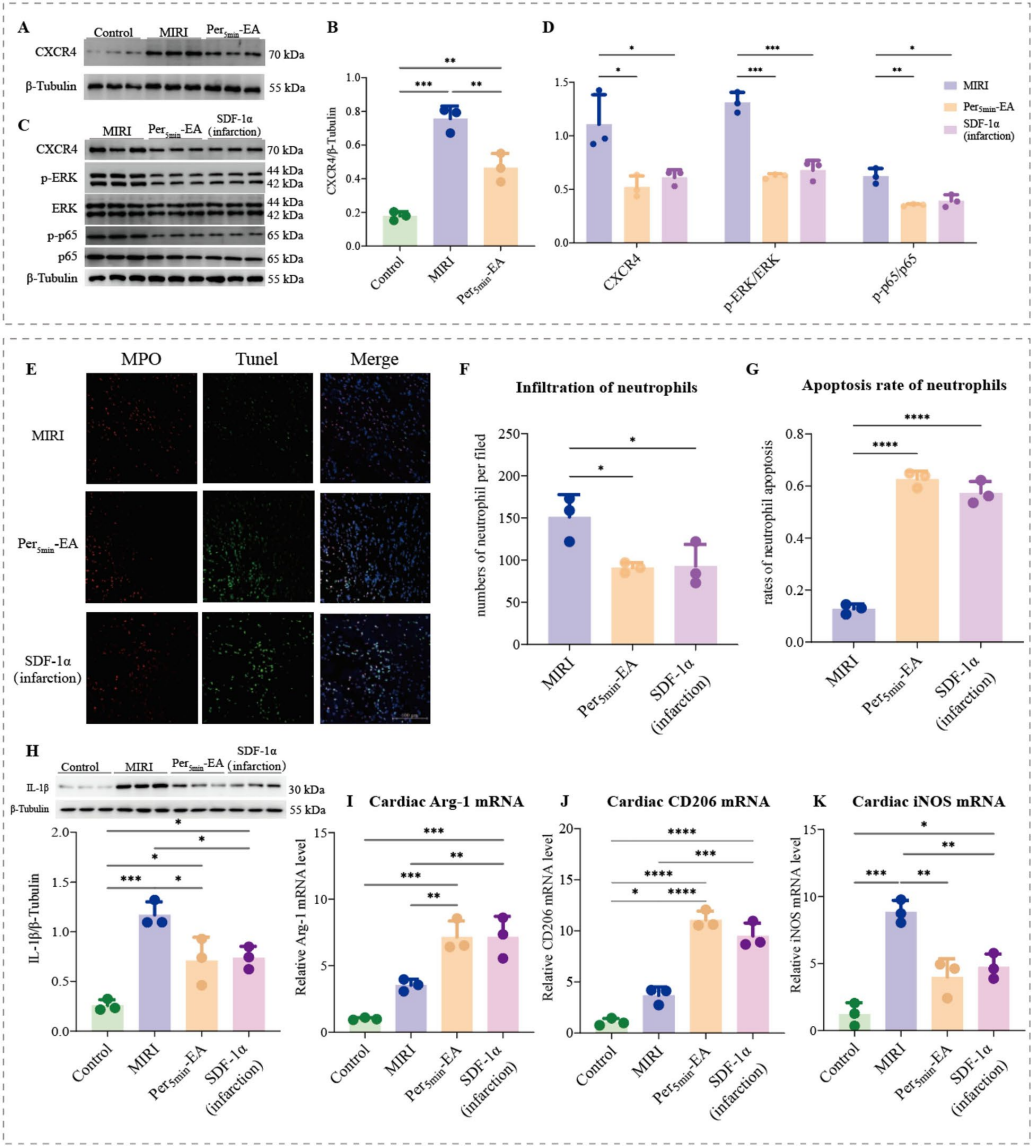
Per_5min_-EA saturates SDF-1α signals to downregulate the CXCR4/ERK/NF-κB pathway, thereby driving neutrophil apoptosis at 24 h post-reperfusion. **A**, **B** Representative Western blot images and quantitative analysis of CXCR4 protein expression in the infarct zone 24 h after MIRI, n = 3. **C**, **D** Representative Western blot images and quantitative analysis of CXCR4, p-ERK/ERK, and p-p65/p65 (NF-κB) ratios in the infarct zone, n = 3. **E** MPO immunofluorescence staining showing neutrophil infiltration in the infarcted myocardium; MPO and TUNEL co-staining identifying apoptotic neutrophils. Red: MPO; green: TUNEL; blue: DAPI. Magnification: × 40. Scale bar = 100 μm. **F** Number of MPO⁺ cells, representing neutrophil accumulation, n = 3. **G** Proportion of MPO⁺TUNEL⁺ cells, indicating apoptotic neutrophils. Apoptosis rate = (number of MPO⁺TUNEL⁺ cells)/(number of MPO⁺ cells), n = 3. **H** IL-1β protein expression in the infarct zone of Per_5min_-EA and SDF-1α (infarction) groups detected by Western blot 24 h after MIRI, n = 3. **I**–**K** mRNA expression of iNOS, CD206, and Arg1 in the infarct zone measured by RT‑qPCR 24 h after MIRI in Per_5min_-EA or SDF-1α (infarction) groups, n = 3. Data are presented as mean ± SD. *p < 0.05, **p < 0.01, ***p < 0.001, **** p < 0.0001

Previous studies indicate that myocardial ischemia upregulates cardiac expression of CXCR4, the receptor for SDF-1α, implicating these factors in the ischemic response [14]. To explore whether CXCR4 contributes to the cardioprotective mechanism of Per_5min_-EA, we assessed its expression in the infarcted myocardium by Western blot. CXCR4 protein levels were increased in the MIRI group but significantly reduced by Per_5min_-EA treatment (Fig. 5A, B), suggesting that downregulation of CXCR4 is associated with the protective effect of Per_5min_-EA after 24 h of MIRI. This result aligns with prior reports that CXCR4 overexpression exacerbates inflammatory injury and apoptosis, worsening cardiac function, whereas CXCR4 inhibition is cardioprotective [26,27]. CXCR4 suppression may promote apoptosis via the RISK pathway [28], potentially explaining the pro-apoptotic effect of Per_5min_-EA on neutrophils.

### Per_5min_-EA suppresses the CXCR4/ERK/NF-κB pathway via saturating signals of SDF-1α

Previous studies indicate that saturating SDF-1α signals promote degradation of approximately 70% of activated CXCR4 receptors [29,30]. Given that Per_5min_-EA significantly elevated SDF-1α levels, we directly injected SDF-1α into the infarct zone to determine whether it is responsible for the Per_5min_-EA–induced suppression of CXCR4. As shown in Fig. 5C, D, the SDF-1α (infarction) group exhibited a significant reduction in CXCR4 expression relative to the MIRI group, an effect comparable to that of Per_5min_-EA. Since CXCR4 is known to activate ERK, leading to NF-κB activation [31], we evaluated this pathway at 24 h post-MIRI. SDF-1α (infarction) markedly suppressed phosphorylation of ERK and NF-κB p65, an effect not significantly different from that of Per_5min_-EA (Fig. 5C,D). These results demonstrate that Per_5min_-EA inhibits the CXCR4/ERK/NF-κB axis via saturating signals of SDF-1α.

### Saturating signaling of SDF-1α is the core mechanism for neutrophil apoptosis induced by Per_5min_-EA

Based on our demonstration that SDF-1α saturating signaling inhibits the CXCR4/ERK/NF-κB pathway after 24 h of MIRI, combined with evidence that suppression of CXCR4 or NF-κB promotes neutrophil apoptosis [26,32], we hypothesize that SDF-1α saturating signaling is the key mechanism by which Per_5min_-EA enhances neutrophil apoptosis after MIRI. To test this, SDF-1α was injected directly into the infarct zone. The results showed that SDF-1α (infarction) significantly increased the neutrophil apoptosis rate and alleviated neutrophil infiltration in the infarcted myocardium, an effect comparable to the Per_5min_-EA group (Fig. 5E–G). Given that Per_5min_-EA sustainably elevates SDF-1α levels in the infarcted myocardium, these findings indicate that SDF-1α saturating signaling is the core mechanism through which Per_5min_-EA promotes neutrophil apoptosis after 24 h of MIRI.

The natural resolution of inflammation begins with neutrophil apoptosis [20], and reduced NF-κB activation also suppresses the expression of inflammatory cytokines such as IL-1β [33]. In the present study, we found that SDF-1α (infarction) replicated the effect of Per_5min_-EA in reducing IL-1β levels (Fig. 5H). Furthermore, apoptotic neutrophils release apoptotic bodies that can reprogram macrophages, promoting the transition from a pro-inflammatory to an anti-inflammatory phenotype [20]. We observed that Per_5min_-EA reduced iNOS and elevated CD206/Arg1 mRNA levels; SDF-1α (infarction) group produced a similar effect (Fig. 5I–K). These results indicate that Per_5min_-EA operates via SDF-1α to suppress IL-1β and drive anti-inflammatory macrophage polarization, thus promoting inflammatory resolution.

### The cardioprotective effect of Per_5min_-EA was not replicated by CXCR4 inhibition

**Fig. 6:**
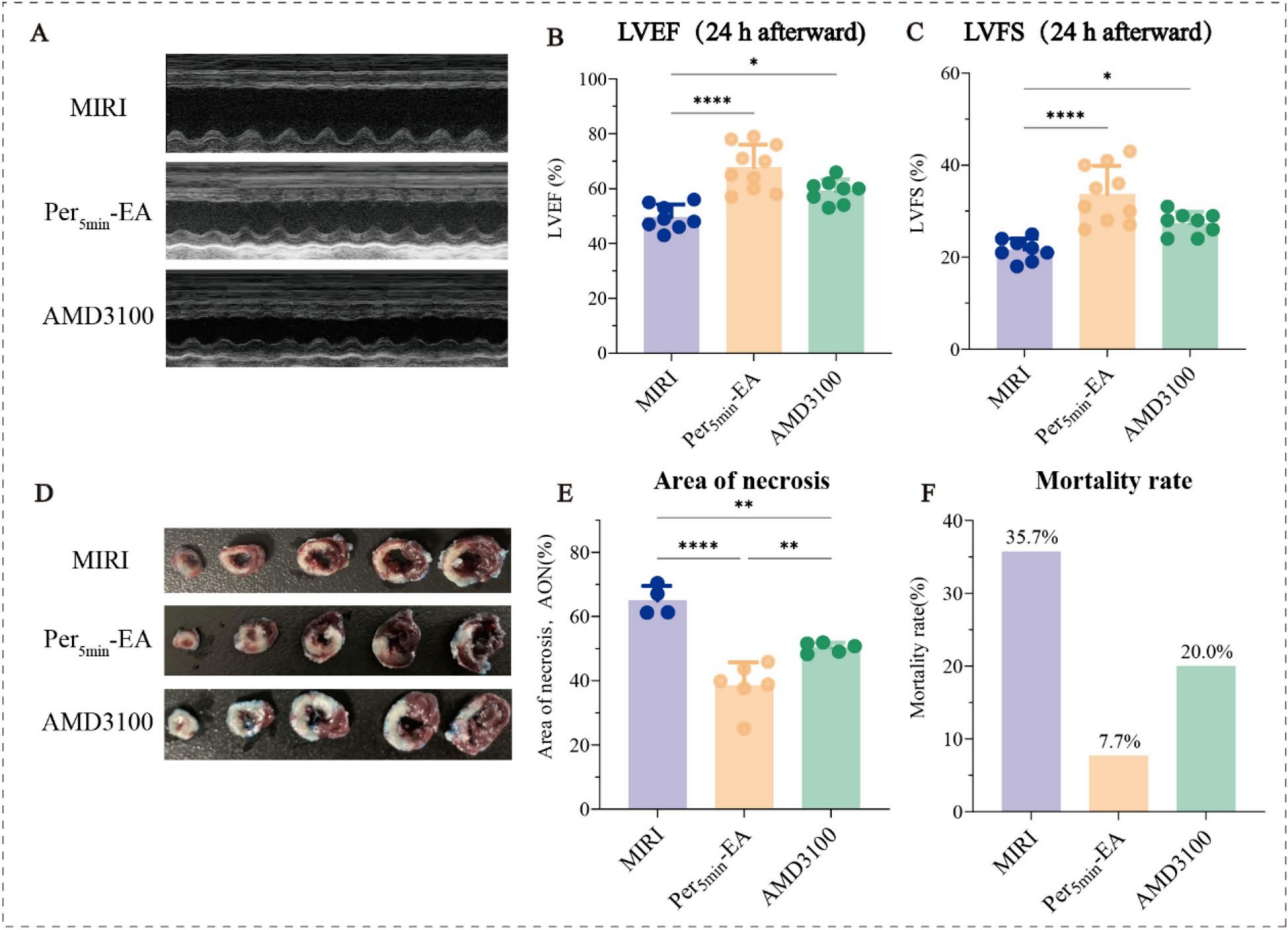
AMD3100 only partially recapitulates the cardioprotection conferred by Per_5min_-EA. **A** Representative M-mode echocardiograms showing left ventricular wall motion at 24 h after MIRI. **B**, **C** LVEF and LVFS measurements from each group, n = 7–10. **D** Representative TTC–Evans blue-stained cross-sections of the left ventricle 24 h after MIRI. Blue: non-ischemic tissue; non-blue: area at risk (AAR); white: necrotic area (AON); red: ischemic but viable myocardium. **E** Infarct size expressed as a percentage of AAR (AON/AAR × 100%), n = 4–6. **F** Mortality rates 24 h after MIRI. All data are presented as mean ± SD. *p < 0.05, **p < 0.01, ***p < 0.001, **** p < 0.0001

The above findings indicate that Per_5min_-EA induces CXCR4 receptor degradation through SDF-1α saturating signaling, thereby inhibiting downstream pathways and exerting cardioprotective effects. To investigate whether Per_5min_-EA confers cardioprotection directly by inhibiting CXCR4, we administered AMD3100 (a CXCR4 antagonist) and evaluated cardiac function and infarct size after 24 h of MIRI. The results demonstrated that AMD3100 significantly improved cardiac function at 24 h post-MIRI, with LVEF and LVFS values of 60 ± 3.79% and 29.50 ± 1.38%, respectively, showing no statistically significant difference compared to the Per_5min_-EA group (Fig. 6 A-C). Meanwhile, AMD3100 also reduced infarct size to 50.19 ± 1.45%, while this value remained elevated compared to the Per_5min_-EA group (Fig. 6 D-E). The AMD3100 group exhibited a higher mortality rate than the Per_5min_-EA group. These results indicate that AMD3100-mediated CXCR4 inhibition confers weaker cardioprotection than Per_5min_-EA; the basis for this difference will be further investigated.

### Sustained SDF-1α upregulation by Per_5min_-EA drives CXCR4 from activation to desensitization and degradation

The CXCR4 receptor exhibits a dual role in MIRI. In isolated cardiomyocytes, SDF-1-induced CXCR4 activation enhances ERK phosphorylation, but this signal rapidly diminishes within 5 min and disappears by 30 min [34,35]. Another study demonstrated that prolonged SDF-1α stimulation for 6 h leads to degradation of nearly 75% of CXCR4 receptors [29,36]. Based on these findings, we hypothesized that Per_5min_-EA promotes sustained SDF-1α expression, driving CXCR4 from initial activation to subsequent desensitization and degradation. Supporting this, ischemic preconditioning has been shown to enhance ERK phosphorylation at 15 min post-I/R, contributing to cardioprotection [37,38].

**Fig. 7:**
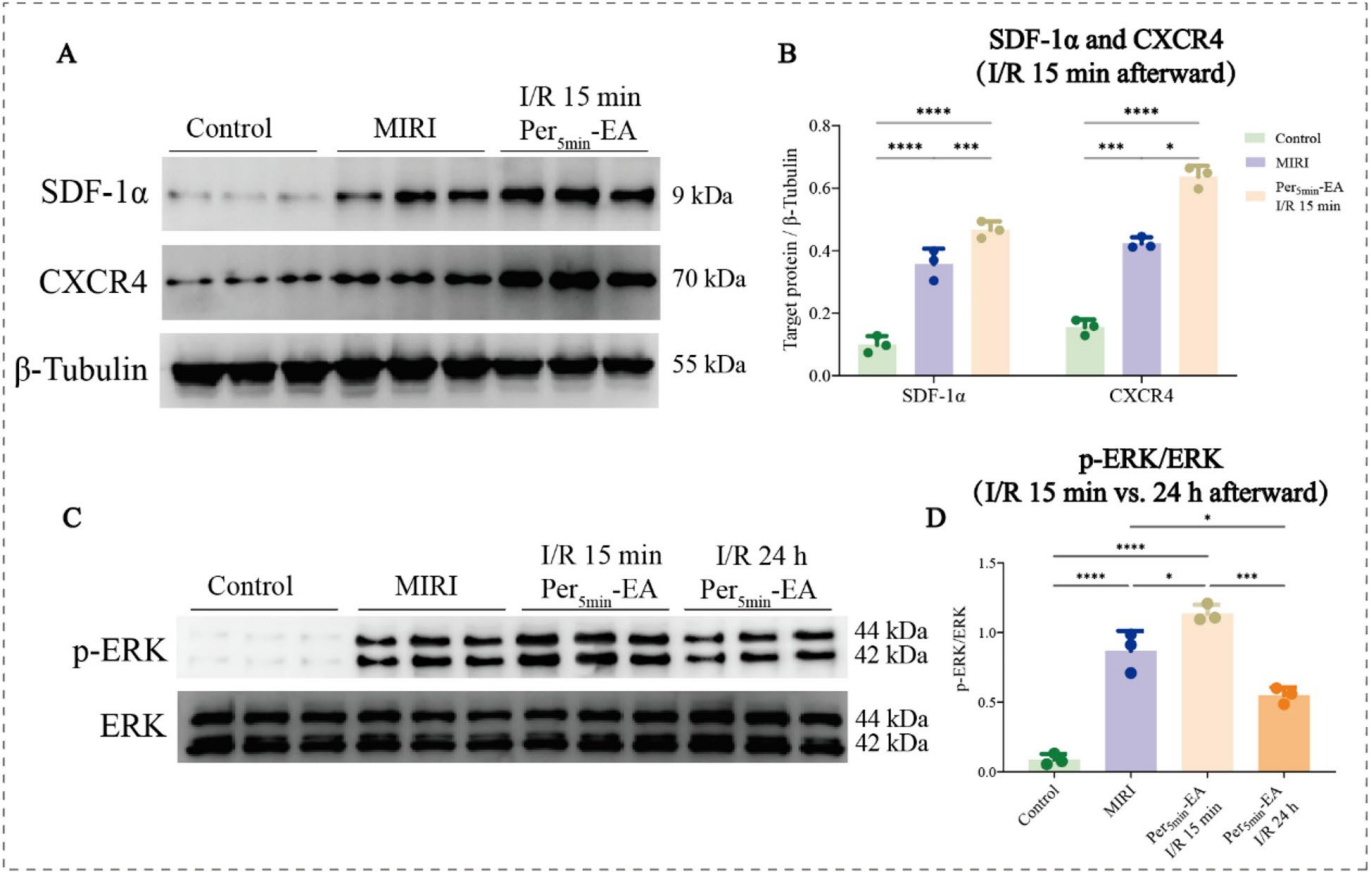
Per_5min_-EA drives the CXCR4 receptor from activation to desensitization and degradation via sustained SDF-1α expression. **A**, **B** Protein levels of CXCR4 and SDF-1α in the infarct zone of the Per_5min_-EA group measured by Western blot 15 min after I/R, n = 3. **C**, **D** Comparison of p-ERK/ERK ratios in the Per_5min_-EA group at 15 min and 24 h after I/R by Western blot, n = 3. All data are presented as mean ± SD. *p < 0.05, **p < 0.01, ***p < 0.001, **** p < 0.0001

To test this hypothesis, we examined SDF-1α, CXCR4 and ERK expression at 15 min after MIRI. At this early time point, Per_5min_-EA significantly increased the expression of SDF-1α and CXCR4, alongside enhanced ERK phosphorylation (Fig. 7A–D). By 24 h post-MIRI, ERK phosphorylation in the Per_5min_-EA group was decreased compared to the 15-min time point (Fig. 7C, D), consistent with our earlier observation of CXCR4 suppression at this later stage (Fig. 5A, B). Notably, Per_5min_-EA elevated SDF-1α expression at both time points (Figs. 7A, B and 4A, B). These results indicate that Per_5min_-EA promotes sustained SDF-1α expression in the infarcted myocardium, dynamically regulating the CXCR4/ERK pathway by activating it at 15 min and promoting its degradation by 24 h post-MIRI.

Reperfusion triggers and accelerates apoptosis [39]. Rapid ERK activation promotes cardiomyocyte survival, an effect not achievable through CXCR4 inhibition alone, explaining why mere CXCR4 blockade fails to replicate the cardioprotection seen with Per_5min_-EA. Thus, the key mechanism underlying Per_5min_-EA's cardioprotection lies in its ability to sustain concentrated SDF-1α expression, leveraging the functional versatility of CXCR4 to produce time-dependent protective effects that align perfectly with the spatiotemporal dynamics of inflammatory responses.

## Disscussion

In this study, we have used MIRI model to investigate the cardioprotection of Per-EA and its role in regulating inflammation resolution following MIRI. We have found that remote EA perconditioning reduces infarct size and improves ventricular function by limiting the post-reperfusion inflammatory response. Mechanistically,​ we identified that SDF-1α acts as a key humoral signal transmitted to the heart in response to EA stimulation. The cardioprotective effect of Per_5min_-EA relies on its ability to concentrate SDF-1α in the infarct zone by enhancing serum levels and inhibiting CXCR7-dependent internalization. SDF-1α orchestrates stage-specific protection: initially activating CXCR4/ERK signaling to inhibit apoptosis during early reperfusion, and subsequently inducing CXCR4 degradation to facilitate neutrophil apoptosis and inflammation resolution. In summary, Per_5min_-EA does not merely suppress inflammation following reperfusion but actively orchestrates its spatiotemporal progression via SDF-1α, guiding it from onset to resolution.

Remote ischemic conditioning (RIC) by brief cycles of limb ischemia and reperfusion displayed a robust cardioprotection against MIRI [40]. However, accumulating evidence suggests that transient ischemia is not a requisite trigger for remote cardioprotection, and several non-ischemic stimuli, including electroacupuncture, have emerged as effective alternatives that recapitulate the cardioprotective effects of RIC [5]. Defined by its non-invasive, non-ischemic, and facile administration, EA stands out as a clinically meaningful and promising green therapy among cardioprotective remote conditioning strategies. The cardioprotective effect of EA at PC6 acupoint on forelimb is well-established, characterized by attenuated I/R injury, reduced myocardial enzyme levels, and a nearly 50% reduction in infarct size [41,42]. In this study, we confirmed that EA significantly improve cardiac function, reduce infarct size, lower myocardial enzyme levels, and decrease mortality.

Uniquely, our protocol initiated EA during late ischemia to mimic the clinical “pre-hospital window”, including ambulance transport or preoperative triage. This “golden window” allows EA, a simple and deployable procedure, to proactively mitigate reperfusion injury triggered by PCI. While immediate EA at ischemia onset increased mortality due to the hyper-acute stress/arrhythmia window [43], delaying intervention by just 5 min dramatically improved survival. This suggests that EA exerts potent cardioprotection once the acute ischemic state stabilizes. Considering real-world transport delays, EA represents a highly feasible and safe clinical intervention when the therapeutic window is appropriately respected.

The cardioprotection elicited by forelimb EA involves humoral factors, such that their transfer to an isolated recipient heart reduces myocardial infarct size [44]. Studies have shown that EA significantly upregulates cardiac stromal cell-derived factor-1α (SDF-1α/CXCL12), an 8 kDa CXC chemokine, thereby protecting myocardium against MI [8]. In fact, SDF-1α is a key cardioprotective humoral factor, with administration in AMI animal models improving left ventricular function and clinical studies associating it with better heart failure outcomes and quality of life in humans [35,45]. Furthermore, SDF-1α is released into the serum by RIC, contributing to its acute cardioprotective effects [45]. Of note, although cardiac SDF-1α levels increase immediately after MI, this elevation is transient [46,47]. In our study, we demonstrated that EA significantly elevated SDF-1α levels in the infarcted myocardium without local synthesis. This enhancement was accompanied by a marked rise in serum SDF-1α, thus identifying SDF-1α as a key humoral factor mediating EA-induced cardioprotection.

In our study, the myocardial infarct zone exhibited a higher SDF-1α concentration than the border zone following EA treatment, a pattern analogous to the elevated SDF-1α levels observed in the ischemic core versus the border zone of the brain after an ischemic insult [48–50]. Subsequent functional investigation confirmed the necessity of this spatial localization, as the cardioprotective effect was only elicited by SDF-1α injection into the infarct zone, not the border zone. Mechanistically, prior research shows that CXCR7 acts as a scavenger to shape the SDF-1α gradient by depleting it in the penumbra to maintain high levels in the core [51]. Corroborating this paradigm, our data revealed that EA elevated CXCR7 expression in the border zone relative to the infarct zone following MIRI, thereby explaining how EA establishes the high SDF-1α concentration in the infarct zone.

The binding of SDF-1α to CXCR4 regulates essential cellular processes—including migration, adhesion and survival. The concentrated SDF-1α in the infarct zone recruits neutrophils, thus ensuring the clearance of necrotic material in the core while preventing their infiltration into viable tissue. During early reperfusion (within 1 h), SDF-1α–CXCR4 binding activates the pro-survival RISK and/or SAFE pathways, thereby inhibiting apoptosis [52]. However, CXCR4 signaling displays a time-dependent, dual role in MIRI. Adenovirus-mediated CXCR4 overexpression prior to ischemia resulted in increased infarct size, worsened cardiac function, and severe inflammatory cell infiltration at 24 h [26,27]. Accordingly, administration of the CXCR4 antagonist AMD 3100 during initial reperfusion (1 h [52], 2 h [53]) provided no acute cardioprotection, whereas a single dose administered after the acute phase conferred a protective effect [15,16]. Together, these findings underscore that within the dynamic context of MIRI, the timing of interventions targeting CXCR4 is critical to their functional outcome.

In the early phase of reperfusion (15 min), our study showed that EA promoted the expression of the CXCR4 receptor, which in turn triggered ERK activation. This rapid ERK activation via SDF-1α/CXCR4 promoted cardiomyocyte survival and attenuated reperfusion-induced apoptosis [54]. On the other hand, prolonged SDF-1α stimulation leads to the degradation of approximately 75% of CXCR4 receptors [29,36]. Correspondingly, we found that at 24 h post-reperfusion, EA downregulated CXCR4 expression, along with phosphorylation of its downstream effectors ERK and NF-κB p65. This coordinated downregulation may facilitate inflammatory resolution by promoting neutrophil apoptosis–a process linked to the inhibition of CXCR4 and NF-κB signaling [26,27], thereby elucidating the mechanism behind EA-induced neutrophil apoptosis at 24 h post-MIRI. Therefore, the cardioprotective effect of EA depends on the dual role of CXCR4 signaling across different temporal windows following MIRI.

**Fig. 8:**
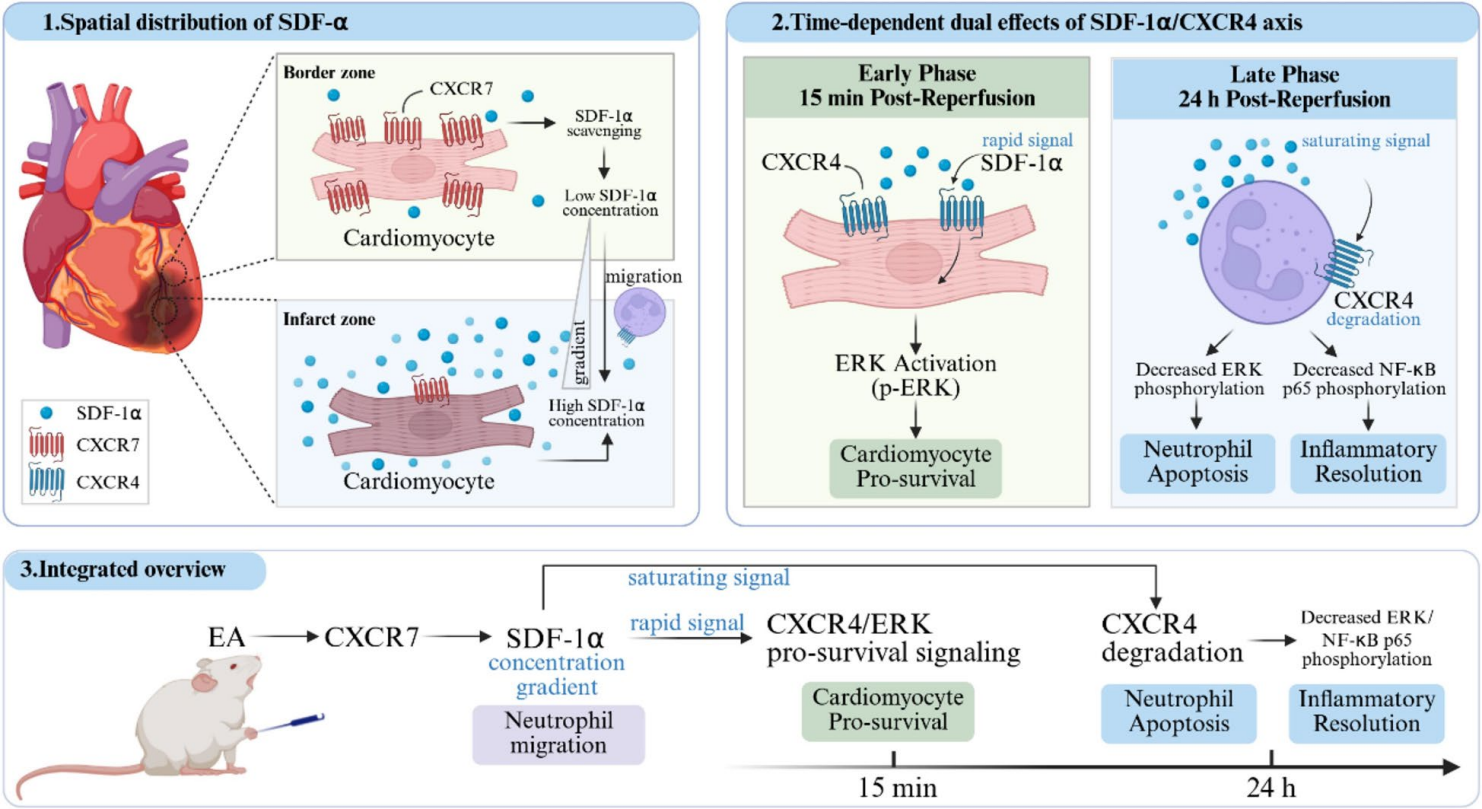
Schematic illustration of the spatiotemporal mechanisms underlying Per_5min_-EA -induced cardioprotection against myocardial ischemia–reperfusion injury

In conclusion, we demonstrate that the cardioprotective effect of remote EA perconditioning is mediated through a spatiotemporally orchestrated mechanism driven by humoral factor SDF-1α. Spatially, the confined enrichment of SDF-1α within the infarct zone by CXCR7 promotes targeted neutrophil recruitment, facilitating necrotic clearance while protecting viable myocardium. Temporally, SDF-1α signaling exerts biphasic regulation: during early reperfusion (15 min), it rapidly activates ERK via CXCR4 to promote cardiomyocyte survival. By 24 h after reperfusion, however, prolonged SDF-1α stimulation leads to CXCR4 degradation, thereby promoting neutrophil apoptosis and facilitating the resolution of inflammation (Fig. 8).

## Data Availability

No datasets were generated or analysed during the current study.

## Abbreviations

AAR: Area at risk
AMI: Acute myocardial infarction
AON: Area of necrosis
Arg1: Arginase 1
CK-MB: Creatine kinase-MB
cTnI: Cardiac troponin I
CXCR4: CXC chemokine receptor type 4
CXCR7: CXC chemokine receptor type 7
DAPI: 4′,6-diamidino-2-phenylindole
EA: Electroacupuncture
ELISA: Enzyme-linked immunosorbent assay
ERK: Extracellular signal-regulated kinase
GAPDH: Glyceraldehyde-3-phosphate dehydrogenase
H&E: Hematoxylin and eosin
IL-1β: Interleukin-1 beta
iNOS: Inducible nitric oxide synthase
I/R: Ischemia/reperfusion
LAD: Left anterior descending coronary artery
LVEF: Left ventricular ejection fraction
LVFS: Left ventricular fractional shortening
MI: Myocardial infarction
MIRI: Myocardial ischemia–reperfusion injury
MPO: Myeloperoxidase
NF-κB: Nuclear factor kappa B
PC6: Pericardium 6 (Neiguan acupoint)
RIC: Remote ischemic conditioning
RISK: Reperfusion injury salvage kinase
SAFE: Survivor activating factor enhancement
SD: Sprague-Dawley
SDF-1α: Stromal cell-derived factor-1 alpha
TNF-α: Tumor necrosis factor alpha
TTC: 2,3,5-Triphenyltetrazolium chloride
TUNEL: Terminal deoxynucleotidyl transferase dUTP nick end labeling
